# Perioperative Management of Polytrauma Patients with Severe Traumatic Brain Injury Undergoing Emergency Extracranial Surgery: A Narrative Review

**DOI:** 10.3390/jcm11010018

**Published:** 2021-12-21

**Authors:** Edoardo Picetti, Israel Rosenstein, Zsolt J. Balogh, Fausto Catena, Fabio S. Taccone, Anna Fornaciari, Danilo Votta, Rafael Badenes, Federico Bilotta

**Affiliations:** 1 Department of Anesthesia and Intensive Care, Parma University Hospital, 43100 Parma, Italy; edoardopicetti@hotmail.com (E.P.); anna.fornaciari@outlook.it (A.F.); 2 Department of Anesthesiology and Critical Care, Policlinico Umberto I Hospital, La Sapienza University of Rome, 00161 Rome, Italy; rosenstein.1867369@studenti.uniroma1.it (I.R.); danilovotta@gmail.com (D.V.); bilotta@tiscali.it (F.B.); 3 Department of Traumatology, John Hunter Hospital, University of Newcastle, Newcastle 2305, Australia; Zsolt.Balogh@health.nsw.gov.au; 4 Department of General and Emergency Surgery, Bufalini Hospital, 47521 Cesena, Italy; faustocatena@gmail.com; 5 Department of Intensive Care, Erasme Hospital, Université Libre de Bruxelles, 1070 Brussels, Belgium; ftaccone@ulb.ac.be; 6 Department of Anesthesiology and Intensive Care, Hospital Clìnico Universitario de Valencia, University of Valencia, 46010 Valencia, Spain

**Keywords:** traumatic brain injury, polytrauma, bleeding, hemorrhagic shock, simultaneous multisystem surgery, anesthesia, perioperative management

## Abstract

Managing the acute phase after a severe traumatic brain injury (TBI) with polytrauma represents a challenging situation for every trauma team member. A worldwide variability in the management of these complex patients has been reported in recent studies. Moreover, limited evidence regarding this topic is available, mainly due to the lack of well-designed studies. Anesthesiologists, as trauma team members, should be familiar with all the issues related to the management of these patients. In this narrative review, we summarize the available evidence in this setting, focusing on perioperative brain protection, cardiorespiratory optimization, and preservation of the coagulative function. An overview on simultaneous multisystem surgery (SMS) is also presented.

## 1. Introduction

Polytrauma (significant injuries of 3 or more points according to the Abbreviated Injury Scale (AIS) in two or more regions in conjunction with one or more additional variables from these five physiologic parameters: systolic blood pressure (SBP) ≤90 mmHg), Glasgow Coma Scale (GCS) score ≤8, base excess (BE) ≤−6.0, international normalized ratio (INR) ≥1.4/partial thromboplastin time (PTT) ≥40 s, and age ≥70 years) is a complication in up to 70% of traumatic brain injury (TBI) cases, which is in itself a leading cause of mortality and disability worldwide [1,2,3,4]. The majority (50–60%) of polytrauma patients die within 4 h following injury (immediate/early peak), either in-scene, in the emergency department (ED), or in the operating room (OR), and among survivors a substantial proportion die within 24 h [3,4,5]. In about half of the patients with severe TBI (GCS ≤ 8), either isolated or in polytrauma, death occurs within 2 h following the injury [6]. A recent multicenter observational study, involving 1536 trauma patients, identified exsanguination as the most frequent cause of early death and TBI as the most common cause of delayed mortality and disability [7].

The first-line approach in polytrauma patients includes hemorrhage control and hemostatic resuscitation, which are recommended treatment strategies that primarily address bleeding and contamination, and include therapies targeting various issues (hemodynamic, ventilatory, and coagulation) [8,9,10,11]. Utilization of simultaneous multisystem surgery (SMS) is gradually becoming recognized as a useful tool in this context [12,13,14]. Some of the treatments utilized to manage polytrauma patients without TBI, including those of the damage control surgery (DCS) and the damage control resuscitation (DCR), are contraindicated when TBI is present, and can impose an additional risk to a patient’s survival and functional outcome [15,16].

Although several studies have addressed the treatment of polytrauma patients and TBI patients independently, there are limited evidence and insights on optimal perioperative management during emergency extracranial surgery (EES), when the two are associated. Moreover, recent studies have demonstrated great variability among trauma centers in classifying, treating, and even studying these complex patients, both between different countries and between regions within the same country [17,18,19,20,21,22]. The objective of this narrative review is to summarize available evidence on perioperative management of polytrauma patients with severe TBI undergoing EES. Differences in treating polytrauma with or without an associated TBI will be highlighted. Aspects related to cardiorespiratory optimization, coagulation (including transfusion) management, and neuroprotection are presented, together with an overview of the emerging role of SMS in this clinical setting.

## 2. Perioperative Cardiorespiratory Optimization

### 2.1. Hemodynamic Management

Exsanguination is the most preventable and the second most common cause of death in polytrauma patients; it is the main target of the hemorrhage control and massive transfusion protocols [7,8,9,10]. An important part of the DCR is the utilization of permissive hypotension (SBP < 90 mmHg) in order to reduce bleeding, which has been known for over a century to help exsanguinating patients [8,10].

Permissive hypotension has been proven to be highly effective in improving outcomes in polytrauma patients without TBI (grade 1 C recommendation), but it should be aggressively avoided in the presence of TBI [23,24]. In this regard, hypotension is recognized as a major secondary cerebral insult when TBI is present; it dramatically increases mortality rate and worsens neurological outcomes in these patients [25,26,27]. Moreover, even a single episode of hypotension (with SBP < 90 mmHg) is associated with a worsened outcome, and each subsequent hypotensive episode has a cumulative effect on mortality risk [28,29,30,31]. In polytrauma patients with TBI, the Brain Trauma Foundation (BTF) guidelines recommend maintaining SBP ≥ 100 mmHg for patients 50–69 years, and ≥110 mmHg for patients 15–49 years or older than 70 years [23]. Furthermore, the European guidelines recommend maintaining a mean arterial pressure (MAP) ≥80 mmHg in polytrauma patients with severe TBI (grade 1C) [24]. Notably, a survey reported that lower thresholds are mis-considered to be safe by 25–56% of acute care surgeons for patients at risk of intracranial hypertension, undergoing EES [18].

In conclusion, in polytrauma patients with TBI, hypotension should be avoided, and it is recommended to maintain SBP > 100 mmHg or MAP > 80 mmHg during EES, as well as during emergency neurosurgery [32].

### 2.2. Respiratory Management

In this section, gas exchange abnormalities (hypoxia, hyperoxia, hypocapnia/hypercapnia) in the management of perioperative EES are described, considering polytrauma patients with and without TBI.

#### 2.2.1. Hypoxemia

Hypoxemia (SpO_2_ < 90% or PaO_2_ < 60 mmHg) frequently complicates trauma scenarios (prehospital incidence rate of ~30%) and should be prevented and treated in all polytrauma patients, with and without TBI [33,34,35,36]. In patients with polytrauma without TBI, a prehospital SpO_2_ < 94% was associated with increased in-hospital mortality and worse disability at discharge (as compared with time of injury) [37]. In those with TBI, even a single episode of SpO_2_ ≤ 92% was associated with increased mortality and disability [26,38,39,40].

#### 2.2.2. Hyperoxemia

Hyperoxemia, especially when extreme (PaO_2_ > 200 mmHg), should also be avoided in polytrauma patients with and without TBI [34,41]. Extreme hyperoxia (PaO_2_ > 200 mmHg being the lower threshold) has been associated with worsen outcomes in TBI patients [34,35,42,43]. These negative effects could be attributed to hyperoxia-induced vasoconstriction (cerebral and coronary) and oxygen free radicals’ toxicity [33,44]. The data were recently partially challenged by two retrospective studies. One study investigated trauma patients without TBI, and reported that hyperoxia (PaO_2_ ≥ 150 mmHg) on hospital admission was associated with reduced in-hospital mortality [44]; the other study investigated TBI patients, and did not find an independent association between hyperoxia (PaO_2_ ≥ 300 mmHg) in the first 24 h following admission and increased in-hospital mortality [36].

More data are necessary on this topic especially regarding the threshold value for hyperoxia which could worsen the outcome.

#### 2.2.3. Hypercapnia/Hypocapnia

Deviations in PaCO_2_ values are tolerated in some trauma scenarios (mainly as part of lung-protective ventilation strategies), but PaCO_2_ abnormalities have been shown to impose a major risk to an injured brain, and thus should be avoided in the acute phase following TBI [16,45,46]. These alterations can result either from trauma, or they can be iatrogenic in intubated and mechanically ventilated patients. Although, in trauma patients without TBI, evidence regarding PaCO_2_ abnormalities is scarce, for patients with TBI, the evidence is sufficient to be included in the BTF guidelines. Regarding TBI, hyperventilation-associated hypocapnia (PaCO_2_ < 35 mmHg) reduces ICP by inducing cerebral vasculature vasoconstriction, but is associated with risk of cerebral ischemia and unfavorable neurological outcomes [45,47,48]. Furthermore, hyperventilation can induce increased airway pressure, thus, compromising venous return and, in cases of hypovolemia that is common in bleeding trauma patients, this can exacerbate arterial hypotension [48]. Hypercapnia (PaCO_2_ > 45 mmHg) can also exacerbate intracranial hypertension, by inducing vasodilation of cerebral vessels and an increase in cerebral blood volume [49]. However, a recent large retrospective study suggested that hypercapnia is dangerous for brain-injured patients and worsened mortality only when associated with acidosis, whereas compensated hypercapnia had no such effect [50]. Ultimately, a PaCO_2_ level of 35–40 mmHg has been recommended in the presence of TBI, with the utilization of transitory hypocapnia only in cases of cerebral herniation confirmed by signs of intracranial hypertension, awaiting or during emergency neurosurgery [32]. Brain oxygen monitoring has been suggested for adjusting PaO_2_ and PaCO_2_ target values, although clear benefit of this method has not been demonstrated [51,52].

## 3. Coagulation (Including Transfusion) Management

In this section, hemoglobin (Hb)-based red blood cells (RBCs) transfusion thresholds, coagulation management, and plasma (P)/platelets (PLTs)/RBCs transfusion ratios in the perioperative management of EES are described, considering polytrauma patients with and without TBI.

### 3.1. Hemoglobin-Based Transfusion Thresholds

Hb value threshold for RBC transfusions in all bleeding traumatic patients should be between 7 and 9 g/dL, according to the European guidelines (grade 1C) [24]. The specific optimal Hb threshold for transfusions in polytrauma patients with TBI is unknown. A recent international survey on Hb values for RBC transfusions in patients with acute brain injury (including TBI) showed that most respondents used an Hb threshold of 7–8 g/dL to initiate transfusion [53]. Trials conducted to compare restrictive (Hb < 7 g/dL) and liberal (Hb < 10 g/dL) transfusion thresholds have shown no difference in mortality and neurological outcomes in polytrauma patients, both with and without TBI [54,55]. Moreover, one trial showed that a liberal transfusion threshold was associated with a higher rate of adverse events during ICU stays [52]. Notably, a recent systematic review and meta-analysis demonstrated that the use of erythropoiesis-stimulating agents was associated with lower mortality in critically ill trauma patients, but no differences in six-month neurological outcomes were detected [56].

During interventions to control life-threatening hemorrhage or emergency neurosurgery, an RBC transfusion is recommended only when Hb drops < 7 g/dL if the patient is hemodynamically stable [32]; this threshold has also been reported in other guidelines and consensus conferences [24,57,58]. If the patient is hemodynamically unstable, or has preexisting cardiovascular diseases, transfusion management should be tailored according to individual needs [32].

In this regard, two ongoing trials are of particular interest. The “Transfusion Strategies in Acute Brain Injured Patients (TRAIN)” study (NCT02968654 on ClinicalTrials.gov) is a large multicenter prospective randomized trial that is comparing a liberal (Hb > 9 g/dL) versus a restrictive (Hb > 7 g/dL) threshold for transfusion in 1000 brain-injured patients. However, this trial includes the exclusion criteria of active hemorrhage at enrolment, and therefore the results might not fully apply to some of the clinical settings discussed in this review. This study is estimated to end in September 2022. Another ongoing trial that is specifically focusing on transfusion practices in TBI patients is the “HEMOglobin transfusion threshold in Traumatic brain Injury OptimizatioN: The HEMOTION Trial” (NCT03260478 on ClinicalTrials.gov), with an estimated time of completion by the end of 2022.

### 3.2. Coagulation Management

Coagulopathy frequently complicates trauma, and, if untreated, it is associated with increased mortality; hence, the utilization of blood products is a key component of DCR [8,10,11,59]. Moreover, in polytrauma patients with TBI, coagulopathy has been associated with further progression of post-traumatic cerebral hematomas and unfavorable neurological outcomes [60,61]. However, large and well-powered studies regarding optimal coagulation management in polytrauma patients with TBI are lacking, and no specific guidelines regarding coagulation management in TBI patients have been published to date.

In polytrauma patients with and without TBI requiring intervention for life-threatening hemorrhage, the maintenance of PLT count > 50,000/mm^3^ is recommended (grade 1C); a higher value (>100,000/mm^3^) is advisable in cases of ongoing bleeding, presence of TBI, or emergency neurosurgery (including ICP probe insertion, grade 2C) [24,32]. In all trauma patients, it is recommended to maintain prothrombin time (PT) and activated PTT (aPTT) within 1.5 the normal value (grade 1C) [24,32].

Point-of-care (POC) tests, such as thromboelastography (TEG) and rotational thromboelastometry (ROTEM), are increasingly utilized for monitoring coagulation functions in bleeding polytrauma patients [24,62]. If available, POC tests are recommended in polytrauma patients with and without TBI to assess and optimize coagulation during interventions for life-threatening hemorrhage or emergency neurosurgery (including ICP probe insertion) [24,32]. Information about specific coagulation deficiencies can be especially useful in cases of PLT dysfunction induced by trauma and/or drugs and in patients taking novel oral anticoagulants (NOACs) [24,62]. Considering the above, TEG and ROTEM could be even more useful in bleeding TBI polytrauma patients [63]. A recent multi-center randomized controlled trial, which included 396 trauma patients who received empiric major hemorrhage protocols (tranexamic acid (TXA), blood components administered at a 1P/1PLT/1 RBC ratio and limited infusion of crystalloid fluids) adjusted according to viscoelastic hemostatic assays or to conventional coagulation tests, showed no difference in outcomes (24 h after injury mortality and free of massive transfusion, 28-day mortality) [64]. Regarding prespecified subgroup analysis, a reduced mortality (needing to be confirmed in future works) was observed in severe TBI patients treated with viscoelastic hemostatic assays.

Early administration (within 3 h from injury) of TXA helps to prevent bleeding in high-risk/actively bleeding patients and is recommended by the European guidelines (grade 1A) without referring specifically to TBI [24]. In polytrauma patients with TBI, TXA is recommended only in mild-moderate TBIs (GCS 9–15). In moderate TBI (GCS 9–13) with preserved pupillary reactivity, early TXA infusion is recommended; in mild TBI (GCS of 14–15) with evidence of bleeding on CT-scan, TXA administration may be beneficial; in severe TBI (GCS 3–8, most patients arriving in the ICU), TXA seems to provide no benefits [65].

The CRASH-3 study, which included 12,737 patients with TBI, showed no benefits related to early administration of TXA, and a non-significant reduction of 28-day head injury-related mortality in patients with isolated TBI [66]. However, a significant reduction in mortality was observed in patients with mild-moderate TBI, but not in those with severe TBI. The authors conclude that TXA was safe in all TBI patients, and that early administration reduced head injury-related death. A later analysis yielded a more personalized approach, which was translated into the recommendations mentioned above [65]. More recently, two studies further underscored the role of TXA in the context of TBI. A randomized controlled trial proved no improvement in neurological outcomes after 6 months in patients with moderate or severe TBI treated with TXA as compared with a placebo [67]. Moreover, a multicenter cohort study found that, in isolated severe TBI, prehospital administration of TXA was associated with increased mortality [68].

### 3.3. Transfusion Ratios

A massive transfusion (generally defined as 10 units of packed RBCs infused within 24 h) is often necessary in the management of bleeding polytrauma patients [69]. In these cases (especially with TBI), it is recommended to use a transfusion protocol with P/PLT/RBC at a ratio of 1:1:1 [24,32]. Subsequently, this ratio may be modified according to laboratory values and POC test results [32].

The Pragmatic Randomized Optimal Platelet and Plasma Ratios (PROPPR) study included 680 trauma patients with major bleeding and was performed to compare transfusion of P, PLTs, and RBCs at a ratio of 1:1:1 versus at a ratio of 1:1:2, and no difference in mortality was observed between the two groups [70]. Moreover, a greater proportion of patients in the 1:1:1 group reached hemostasis, and fewer patients died due to exsanguination within the first 24 h after injury.

## 4. Perioperative Brain Protection

### 4.1. Intracranial Pressure (ICP)/Cerebral Prefusion Pressure (CPP) Monitoring in Polytrauma Patients with TBI

In patients with TBI, both intracranial hypertension and low CPP (CPP = MAP − ICP) are dangerous; they are potentially associated with worsening of secondary brain damage and can lead to increased mortality and higher level of disability [25,71,72,73]. In comatose patients due to TBI with radiological signs of intracranial hypertension, following exclusion/control of life-threatening hemorrhage, invasive ICP monitoring should be considered, regardless of the need in EES [32].

Episodes of intracranial hypertension and low CPP have been observed during extracranial surgery, when performed within 2 weeks of trauma in patients with concomitant TBI [74,75]. These episodes should be considered in terms of intensity and duration; invasive ICP monitoring is potentially useful for their timely estimation and management, and should be considered according to the BTF guidelines “to reduce in-hospital and 2-week post-injury mortality” (level 2B) [23,72,73,76,77,78]. A recent prospective multicenter study showed that the use of ICP monitoring might be associated with a more intensive therapeutic approach and with a lower six-month mortality in more severe cases [79]. Regarding indications for ICP monitoring, two consensus conferences recommend ICP to be monitored in all salvageable comatose patients with radiological signs of intracranial hypertension, and not to be monitored in patients with minimal intracranial pathology (i.e., diffuse axonal injury and small petechiae) [80,81]. Specifically, Stocchetti et al. recommended ICP monitoring for TBI comatose patients with brain contusions in whom the interruption of sedation to evaluate the neurological status was considered to be dangerous, such as in patients with radiological signs of intracranial hypertension, severe respiratory failure, or ongoing EES [80].

A recent survey showed that most centers insert an ICP monitor in patients with severe TBI and head CT abnormalities, but there was a lack of consensus on other indications (e.g., moderate TBI with contusions, intraventricular hemorrhage, etc.) [17]. However, specific indications regarding which patients’ ICP/CPP should be monitored are missing, due to lack of evidence-based data [23,82]. It is relevant to emphasize that invasive intracranial devices increase the risk for brain infections (i.e., ventriculitis and meningitis); this risk is already higher following trauma [83,84]. However, a lower rate of complications is associated with intraparenchymal probes as compared with ventricular drainage systems [83,84].

ICP monitoring during EES, although recognized as a useful tool, is utilized routinely only in a minority of cases; even protocols are mostly unavailable in this setting [18]. In this regard, concurrent bleeding control and ICP monitoring has been shown to be feasible utilizing a “hybrid emergency room system” (HERS) [85]. The adherence to the abovementioned BP targets is of paramount importance considering that most TBI patients undergoing EES for life-threatening bleeding control do not have ICP monitoring [18].

### 4.2. Perioperative ICP and CPP Management

**Figure 1:**
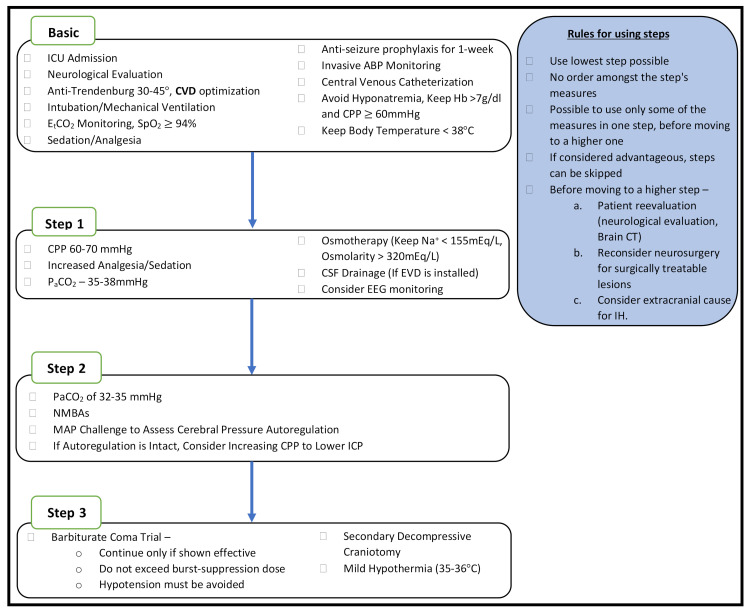
Intracranial pressure monitor-based management algorithm for severe traumatic brain injury patients (modified from Hawryluk GWJ et al. [57]). ICU, intensive care unit; CVD, cerebral venous drainage; EtCO_2_, end tidal carbon dioxide; SpO_2_, arterial oxygen saturation; ABP, arterial blood pressure; CPP, cerebral perfusion pressure, PaCO_2_, arterial partial pressure of carbon dioxide; CSF, cerebral spinal fluid; EVD, external ventricular drain; EEG, electroencephalogram; NMBA, neuromuscular blocking agent; MAP, mean arterial pressure; ICP, intracranial pressure; CT, computed tomography; IH, Intracranial hypertension.

Several recommendations have been published with a focus on ICP/CPP management in adult polytrauma patients with severe TBI, with the aim of establishing a common practice [32]. The following aspects have been considered: (1) osmotherapy and temporary hypocapnia in cases of cerebral herniation, awaiting or during emergency neurosurgery; (2) maintenance of CPP ≥ 60 mmHg; and (3) utilization of a stepwise approach to treat intracranial hypertension (when targeting the underlying pathophysiologic mechanism of elevated ICP is not possible) [86]. According to this approach, the level of intervention is gradually increased, reserving more aggressive procedures (generally associated with greater risks/adverse effects) for situations when no response to previous treatment is observed [87]. A recently proposed management algorithm, regarding adult patients with severe TBI, supports this approach (Figure 1) [57]. In addition, advanced neuromonitoring (i.e., brain tissue oxygenation), generally not available in the very acute phase (first 24 h after admission), may contribute to the personalization of therapy in complex cases, but carries the risk of implicit drawbacks, information overload, and misleading details [51,87,88].

### 4.3. Brain Trauma Foundation (BTF) Guidelines for the Management of Severe TBI

The recommendations of the last BTF guidelines for the management of severe TBI [23,89] can be summarized as follows:

- The use of information from ICP monitoring is recommended to reduce in-hospital and two-week post-injury mortality (LEVEL II B).
- Treat patients with ICP > 22 mmHg is recommended because values above this level are associated with increased mortality (LEVEL II B).
- CPP monitoring is recommended to decrease two-week mortality (LEVEL II B).
- CPP value for survival and favorable outcomes is 60–70 mmHg depending upon the autoregulatory status of the patient (LEVEL II B).
- Avoid aggressive attempts to maintain CPP > 70 mmHg with fluids and pressors should be considered because of the risk of adult respiratory failure (LEVEL III).
- Jugular bulb monitoring of arteriovenous oxygen content difference (AVDO_2_), as a source of information for management decisions, may be considered to reduce mortality and improve outcomes at 3 and 6 months post-injury (LEVEL III).
- Avoid jugular venous saturation <50% to reduce mortality and improve outcomes (LEVEL III).
- SBP ≥ 100 mmHg for patients 50 to 69 years old or≥ 110 mmHg or above for patients 15 to 49 or >70 years old may be considered to decrease mortality and improve outcomes (LEVEL III).
- A combination of ICP values and clinical and brain CT findings may be used to make management decisions (LEVEL III).
- Prophylactic hypothermia is not recommended to improve outcomes in patients with diffuse injury (LEVEL II B).
- Secondary decompressive craniectomy (DC) performed for late refractory ICP elevation is recommended to improve mortality and favorable outcomes (LEVEL II A).
- Secondary DC performed for early refractory ICP elevation is not recommended to improve mortality and favorable outcomes (LEVEL II A).
- A large frontotemporoparietal DC (not less than 12 × 15 cm or 15 cm in diameter) is recommended to reduce mortality and to improve neurological outcomes in patients with severe TBI (LEVEL II A).
- Secondary DC, for early or late refractory ICP elevation, is suggested to reduce ICP and the duration of intensive care, although the relationship between these effects and a favorable outcome is uncertain (LEVEL II A).
- An external ventricular drain (EVD) system zeroed at the midbrain with continuous drainage of cerebrospinal fluid (CSF) may be considered to lower ICP burden more effectively than intermittent use (LEVEL III).
- The use of CSF drainage to lower ICP in patients with an initial GCS < 6 during the first 12 h after injury may be considered (LEVEL III).
- Antimicrobial-impregnated catheters may be considered to prevent catheter-related infections during EVD (LEVEL III).
- Prolonged prophylactic hyperventilation with PaCO_2_ ≤ 25 mmHg is not recommended (LEVEL II B).
- Administration of barbiturates to induce burst suppression as prophylaxis against the development of intracranial hypertension is not recommended (LEVEL II B).
- High-dose barbiturate administration is recommended to control elevated ICP refractory to maximum standard medical and surgical treatment. Hemodynamic stability is essential before and during barbiturate therapy (LEVEL II B).
- Although propofol is recommended for the control of ICP, it is not recommended for improvement in mortality or six-month outcomes (LEVEL II B).
- The use of steroids is not recommended for improving outcomes or reducing ICP (LEVEL I).
- Feeding patients to attain basal caloric replacement at least by the fifth day and, at most, by the seventh day post-injury is recommended to decrease mortality (LEVEL II A).
- Transgastric jejunal feeding is recommended to reduce the incidence of ventilator-associated pneumonia (VAP) (LEVEL II B).
- Early tracheostomy is recommended to reduce mechanical ventilation days when the overall benefit is thought to outweigh the complications associated with such a procedure. However, there is no evidence that early tracheostomy reduces mortality or the rate of nosocomial pneumonia (LEVEL II A).
- The use of povidone-iodine oral care is not recommended to reduce VAP and may cause an increased risk of acute respiratory distress syndrome (ARDS) (LEVEL II A).
- Low-molecular-weight heparin (LMWH) or low-dose unfractionated heparin may be used in combination with mechanical prophylaxis; however, there is an increased risk for expansion of intracranial hemorrhage (LEVEL III).
- In addition to compression stockings, pharmacologic prophylaxis may be considered if the brain injury is stable and when the benefit is considered to outweigh the risk of increased intracranial hemorrhage (LEVEL III).
- There is insufficient evidence to support recommendations regarding the preferred agent, dose, or timing of pharmacologic prophylaxis for deep vein thrombosis (LEVEL III).
- Prophylactic phenytoin or valproate are not recommended for preventing late post-traumatic seizures (PTS) (LEVEL II A).
- Phenytoin is recommended to decrease the incidence of early PTS (within 7 days of injury), when the overall benefit is thought to outweigh the complications associated with such treatment. However, early PTS have not been associated with worse outcomes (LEVEL II A).
- At the present time, there is insufficient evidence to recommend levetiracetam as compared with phenytoin regarding efficacy in preventing early post-traumatic seizures and toxicity (LEVEL II A).

## 5. Simultaneous Multisystem Surgery (SMS)

Hemodynamically unstable polytrauma patients with TBI may require SMS performed by different surgical teams, which aim to control bleeding, contamination, and restore perfusion of critically ischemic organs during the intracranial procedure [12,13,14]. For these situations, due to the lack of high-level evidence, SMS has been recommended based on expert consensus opinion [32].

SMS can be performed in a standard operating room [12,90] or by utilizing a hybrid-emergency room system (HERS) where diagnostic procedures (computed tomography (CT)-scan, echography) and damage control interventions. (angioembolization, resuscitative endovascular balloon occlusion of the aorta (REBOA), DCS, neurosurgery, etc.) are done simultaneously without patient transfer [14,91,92].

The data suggest that the HERS approach has been associated with shorter time to initiate CT scanning, emergency surgery, and fewer unfavorable outcomes in polytrauma patients with and without TBI [13,14,91,92]. A higher survival benefit has been observed particularly in patients with higher ISS or with active bleeding [92]. The use of HERS has also been shown to be cost-effective [93].

While the ability to perform SMS seems to be straightforward and beneficial, only a few centers are equipped and have trained persons to perform it in the context of TBI complicated by polytrauma [18]. This approach, frequently utilized in war scenarios, requires protocols and collaboration between different surgical teams [12,94]. Training and simulation have been proposed as a fundamental tool for HERS performance [94,95]. The Japanese Association for Hybrid Emergency Room System was recently created to specifically address the needs of specialized HERS trauma teams (composed of trauma surgeons, radiologists, anesthesiologists, nurses, etc.) with the main goal to standardize protocols and to promote education [96]. In situations where multiple treating teams need to work together, the role of the trauma team leader is fundamental [94,95]. Communication regarding patient positioning and optimization of access, for all the teams involved, to their respective fields of work, without any dangerous interference and potential cross-contamination, is essential [12,90]. Theoretical and practical training using a simulated environment is essential for all members of the team [94,96].

The CT scanner used for the HERS procedures must be a dedicated one, in order to secure the flow of other trauma and non-trauma emergency patients through this essential imaging modality [94,96]. Furthermore, for preparedness for multiple patients needing HERS, a dual room angio-CT system has been implemented in some hospitals with a mobile CT scanner that can be moved between two shock rooms as needed [96].

The benefits of a hybrid operating room are very difficult to show, due to the diversity of injury combinations among complex polytrauma patients, which is the obvious hurdle to perform randomized controlled studies in this environment and apparent publication bias. Many of the publications in this area have inherited all the flaws of the pre- and post-type studies, but certainly this novel care provision has promising results in terms of timely hemorrhage control in large volume trauma centers, with less blood and factor usage, less nosocomial infections, and fewer days on a ventilator without change in mortality [97].

## 6. Discussion

This narrative review originally summarizes the evidence on perioperative management of polytrauma patients with severe TBI, undergoing extracranial surgery. Perioperative treatment of these patients should focus on preventing secondary brain damage; hence, it differs from treatment of polytrauma patients without TBI in some respects, while it overlaps in other respects. The most notable distinction relates to hemodynamic management; in polytrauma patients with moderate-severe TBI, “permissive arterial hypotension” should be aggressively avoided, while this strategy is highly recommended in polytrauma patients without TBI. A recent study showed that positive fluid balances were associated with worse outcomes in TBI patients, suggesting that normovolemia should be the target in this setting [98]. In this regard, the utilization of advanced hemodynamic monitoring to assess cardiac output or fluid responsiveness (i.e., stroke volume variation, pulse pressure variation, etc.) could be useful in the perioperative period [99]. However, more studies on the use of these forms of monitoring in polytrauma TBI patients are needed.

Optimal respiratory targets (PaO_2_ and PaCO_2_) in polytrauma patients with TBI constitute a field of limited and partially conflicting evidence. However, further research is required to elucidate the effect of hyperoxia and compensated hypercapnia on the injured brain; moreover, a standardized threshold of hyperoxia should be investigated and established. The variables that seem to be similarly managed in all trauma patients are aggressive prevention of hypoxia, Hb-based and coagulation-based transfusion thresholds, transfusion ratios, and criteria for POC use. It should be noted that a higher PLT count than usual is recommended for emergency neurosurgery. The use of TXA is only recommended in selected polytrauma scenarios when associated with TBI. Regarding neuroprotection (relevant only in TBI), intracranial hypertension and low CPP have to be avoided (see Table 1), and ICP monitoring should be considered in all comatose TBI patients with signs of elevated ICP, regardless of the need in EES. Finally, the implementation of SMS in trauma centers is recommended, as well as further research in this field.

In the rapidly growing field of polytrauma patients with TBI, the importance of up-to-date information based on clinical trials and literature analyses cannot be underestimated. Guidelines and common practices are often based on old literature, and articles reporting recent advances and insights in the field have an important contribution [100]. Since guidelines in this field were published several years ago, we consider this review to be an important collection of the new information introduced after the guidelines were publication.

The discussion about a specific approach for treating these patients has reached a higher priority level in recent years, and trauma-focused studies are increasingly addressing patients with TBI as a distinct group with particular needs. Similarly, TBI-focused studies are also considering the context of polytrauma and associated conditions (e.g., bleeding and coagulopathy). Moreover, there is now particular interest in the acute phase management of trauma patients (first 24 h), not only in the context of the primary injury but also in order to target and prevent secondary (and late) injuries [23,24,30,32,51,57,101].

Geeraerts et al. updated the French guidelines of acute phase management of severe TBI and dedicated a section to perioperative treatment of polytrauma patients with TBI [30]. They highlighted the importance of further research to better characterize the relationship between systemic conditions (e.g., MAP and hypoxia) and secondary cerebral insult. Hardcastle et al. [16] dealt with the ventilatory aspect of trauma patients, with special emphasis on the first 24 h and perioperative environment. The authors emphasized that focusing on the phase of injury was of essential importance, due to tissue plasticity, and that implications of acute-phase homeostasis alterations were of major potential.

However, despite the emerging interest in this field, several studies have addressed major incompliances with published guidelines and consensuses on this topic [17,18,19]. A better adherence to protocols manifests in better survival and neurological outcomes [102]. The reasons for non-adherence to guidelines suggest that the poor evidence level in the field of TBI is the major underlying cause [19]. This can be another catalyst for further high-quality clinical research in this field, which is highly needed. Nevertheless, better adherence to protocols and guidelines should be intensively promoted.

We used online databases (PubMed, Google, etc.) for the collection of information. Due to the diverse nature of polytrauma injuries and publications in the field, we used multiple search terms and filter options, mostly focusing on data published in the last 10–15 years. The terms “anesthesia”, “TBI”, “polytrauma”, “acute phase”, “management”, “transfusion”, and several others were used in a variety of combinations. The reason for this study being a narrative review rather than a systematic review is due to the lack of evidence and data. We had to collect data from several fields and build the structure presented in this paper. We strongly recommend further systematic research in the field of polytrauma when associated with TBI.

Ultimately, the results presented in this review suggest that a specific focus should be put on polytrauma patients with TBI. This is true both at the level of trauma centers, in which better implementation of current guidelines should be promoted, and at the level of clinical trials and reviews, which are essential to better elucidate some of the debated issues in the field. We consider of special interest, and highly recommend, further investigation of the seemingly overlapping guidelines for all patients with polytrauma, both with and without TBI (e.g., Hb-level transfusion threshold, coagulopathy-related values, and transfusion ratios). Additionally, the relation between the injured brain and respiratory deviations, as well as the role of ICP/CPP monitoring during EES, should be better established. The utilization of accepted paradigms with insufficient evidence support might turn out to be harmful for these vulnerable patients.

The studies, listed in our references, that relate specifically to polytrauma patients with TBI [12,13,14,18,32,74,75,85,90,91,92,93,94,95,96,97] are mainly written by acute care surgeons with respect to neurosurgeons and anesthesiologists (Table 1). In this regard, a multidisciplinary approach (involving doctors with different specialties) is desirable in the future in order to improve patient outcomes in this difficult setting.

Some limitations might render this review to be less than optimal, especially considering that it is a narrative review. First, only studies published in English were selected. This strategy might omit important data published in other languages. However, since most scientific papers are published in English, and since any novelty in the field would have probably drawn the attention of the scientific community, we do not consider this limitation to be too significant. Second, the nature of a narrative review might suggest a selection bias in selecting the articles discussed in the review. In this regard, we have focused on presenting the most up-to-date data, including large-scale trials and surveys, well-respected guidelines, and well-based consensuses.

## 7. Conclusions

**Figure 2:**
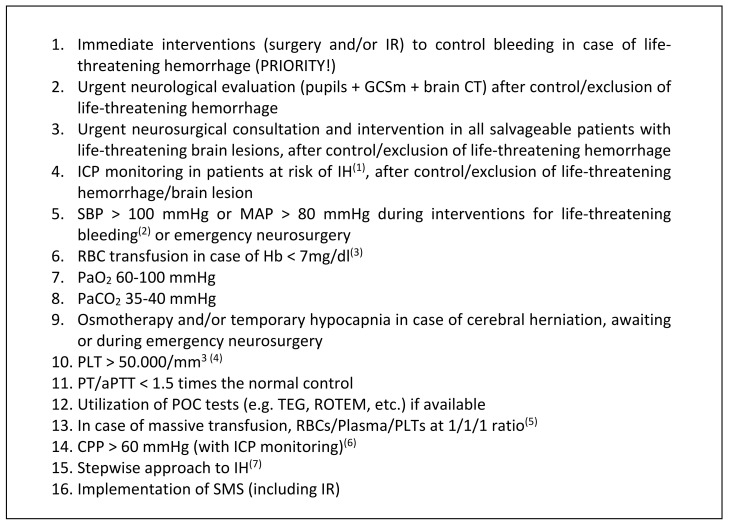
Summary of the World Society of Emergency Surgery consensus conference recommendations, regarding the monitoring and management of severe adult traumatic brain injury patients with polytrauma in the first 24 h (modified from Picetti et al. [32]). ^(1)^ Coma plus radiological signs of IH. ^(2)^ Temporarily lower values in case of difficult bleeding control. ^(3)^ Higher threshold in patients at “risk” (e.g., elderly, pre-existing heart disease, etc.). ^(4)^ Higher values for emergency neurosurgery (including ICP probe insertion). ^(5)^ Later on, the ratio may be modified according to laboratory values. ^(6)^ This value can be personalized considering neuromonitoring data and cerebral autoregulation status. ^(7)^ If impossible to target the underling pathophysiology of IH.

**Figure 3:**
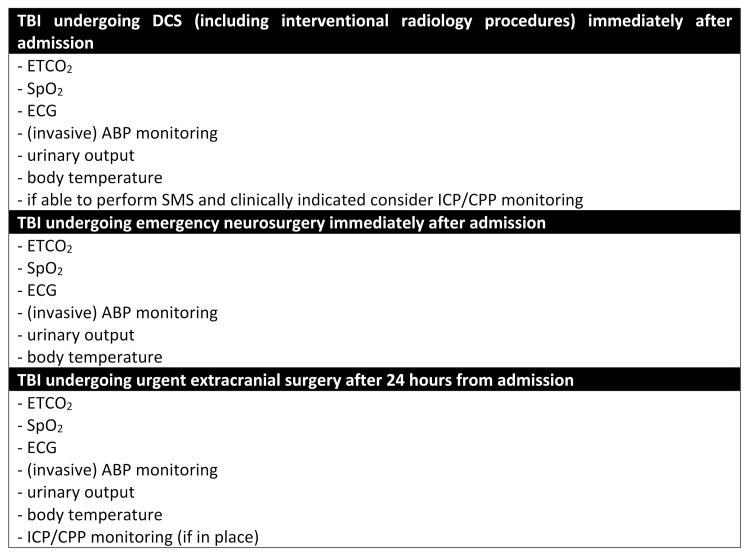
Suggested monitoring for severe TBI patients with polytrauma undergoing surgery. TBI, traumatic brain injury; DCS, damage control surgery; ETCO_2_, end-tidal carbon dioxide; SpO_2_, peripheral oxygen saturation; ECG, electrocardiogram; ABP, arterial blood pressure; ICP, intracranial pressure; CPP, cerebral perfusion pressure; SMS simultaneous multisystem surgery.

Acute phase management of polytrauma patients with severe TBI is a challenging condition, especially in cases of severe cardiorespiratory instability. In addition, most of the monitoring and treatment strategies utilized in these patients are not supported by well-designed studies. In this regard, in Figure 2, we summarized the World Society of Emergency Surgery (WSES) consensus conference guidelines on monitoring and the management of severe adult traumatic brain injury patients with polytrauma in the first 24 h [32]; in Figure 3, we reported our suggestions regarding the monitoring of severe TBI patients with polytrauma undergoing surgery. Anesthesiologists, as well as other members of the trauma team, should know all the problems associated with these patients, and be trained in working within a group. Our aim is that this review can be useful for daily anesthesia practice in this difficult scenario, and will stimulate research in this field.

## Figures and Tables

**Table 1:** Relevant studies regarding polytrauma TBI patients.

Reference Number	Title	Journal	Year
[74]	Intraoperative Secondary Insults during Extracranial Surgery in children with Traumatic Brain Injury	*Child’s Nervous System*	2014
[95]	The Evolution of a Purpose Designed Hybrid Trauma Operating Room from the Trauma Service Perspective: The RAPTOR (Resuscitation with Angiography Percutaneous Treatments and Operative Resuscitations)	*Injury*	2014
[12]	Simultaneous Multisystem Surgery: An Important Capability for the Civilian Trauma Hospital	*Clinical Neurology and Neurosurgery*	2016
[75]	Intraoperative Secondary Insults during Orthopedic Surgery in Traumatic Brain Injury	*Journal of Neurosurgical Anesthesiology*	2017
[13]	Effect of the Hybrid Emergency Room System on Functional Outcome in Patients with Severe Traumatic Brain Injury	*World Neurosurgery*	2018
[85]	First Clinical Experiences of Concurrent Bleeding Control and Intracranial Pressure Monitoring Using a Hybrid Emergency Room System in Patients with Multiple Injuries	*World Journal of Emergency Surgery*	2018
[96]	The Hybrid Emergency Room System: A Novel Trauma Evaluation and Care System Created in Japan	*Acute Medicine & Surgery*	2019
[94]	Simultaneous Damage Control Surgery and Endovascular Procedures for Patients with Blunt Trauma in the Hybrid Emergency Room SYSTEM: New Multidisciplinary Trauma Team Building	*Journal of Trauma and Acute Care Surgery*	2019
[91]	The Survival Benefit of a Novel Trauma Workflow that Includes Immediate Whole-body Computed Tomography, Surgery, and Interventional Radiology, All in One Trauma Resuscitation Room: A Retrospective Historical Control Study	*Annals of Surgery*	2019
[18]	Preserve Encephalus in Surgery of Trauma: Online Survey (P.E.S.T.O)	*World Journal of Emergency Surgery*	2019
[32]	WSES Consensus Conference Guidelines: Monitoring and Management of Severe Adult Traumatic Brain Injury Patients with Polytrauma in the First 24 Hours	*World Journal of Emergency Surgery*	2019
[14]	A Prospective Evaluation of the Utility of a Hybrid Operating Suite for Severely Injured Patients: Overstated or Underutilized?	*Annals of Surgery*	2020
[93]	Cost-Effectiveness of a Hybrid Emergency Room System for Severe Trauma: A Health Technology Assessment from the Perspective of the Third-Party Payer in Japan	*World Journal of Emergency Surgery*	2021
[97]	Clinical Impact of a Dedicated Trauma Hybrid Operating Room	*Journal of the American College of Surgeons*	2021
[92]	Hybrid Emergency Room Shows Maximum Effect on Trauma Resuscitation When Used in Patients with Higher Severity	*Journal of Trauma and Acute Care Surgery*	2021

## Data Availability

No new data were created or analyzed in this study. Data sharing does not apply to this article.

## Abbreviations

IR	Interventional Radiology
GCSm	Glasgow Coma Scale motor score
CT	Computed Tomography
ICP	Intracranial Pressure
IH	Intracranial Hypertension
SBP	Systolic Blood Pressure
MAP	Mean Arterial Pressure
RBC	Red Blood Cell
Hb	Hemoglobin
PaO_2_	Arterial Partial Pressure of Oxygen
PaCO_2_	Arterial Partial Pressure of Carbon Dioxide
PLT	Platelet
PT	Prothrombin Time
aPTT	Activated Partial Thromboplastin Time
POC	Point-Of-Care
TEG	Thromboelastography
ROTEM	Rotational Thromboelastometry
CPP	Cerebral Perfusion Pressure
SMS	Simultaneous Multisystem Surgery
